# E-cadherin-mediated neighborhood surveillance dictates pre-malignant outcomes

**DOI:** 10.64898/2026.09.02.748765

**Published:** 2026-09-04

**Authors:** Elizabeth A.N. Thompson, Clara Schimmer, Tatiana Omelchenko, Michele Paganini, Jesse S.S. Novak, Sienna T. Muller, Alain R. Bonny, Marina Schernthanner, Sairaj M. Sajjath, Charlotte J. Bell, Alp Gorgulu, S. Martina Parigi, Katherine S. Stewart, Priyam Banerjee, Elaine Fuchs, Stephanie J. Ellis

## Abstract

Stratified epithelia accumulate oncogenic mutations throughout life, yet overgrowths are rare. How epithelia detect and eliminate aberrant clones remains poorly understood. Using a mouse model of oncogenic clonal mosaicism in the skin, we find that pre-malignant epidermal cells redistribute E-cadherin to interfaces shared with wild-type neighbors, generating local tension heterogeneity that triggers elimination by cell competition. We show that gain or loss of E-cadherin can each drive competitive elimination, and although mechanical routes differ, both establish tension heterogeneity between neighbors, rather than any absolute adhesion state, as the critical determinant of epidermal fitness. This mechanism carries the seeds of its own failure: these tension differentials precipitate clonal sorting, depleting the wild-type contacts that surveillance requires. Pre-malignant cells then become supercompetitors, eliminating wild-type neighbors and expanding hyperplastically. Mechanical heterogeneity therefore endows tissues with an active, yet inherently fragile error-correction system whose collapse initiates a switch in competitive status, increasing tumorigenesis susceptibility.

## Full Text Availability

The license terms selected by the author(s) for this preprint version do not permit archiving in PMC. The full text is available from the preprint server.

